# E. coli Pyogenic Ventriculitis: A Comprehensive Case Report

**DOI:** 10.7759/cureus.86202

**Published:** 2025-06-17

**Authors:** Matthew A Hibdon, Keri K Allen, Alan Wang, John Greene

**Affiliations:** 1 Osteopathic Medicine, Nova Southeastern University Dr. Kiran C. Patel College of Osteopathic Medicine, Clearwater, USA; 2 Morsani College of Medicine, University of South Florida Morsani College of Medicine, Tampa, USA; 3 Internal Medicine, Moffitt Cancer Center, Tampa, USA

**Keywords:** e. coli bacteremia, pelvic radiation, sigmoidal fistula, ventriculitis, vertebral osteomyelitis

## Abstract

Pyogenic ventriculitis is a rare complication of bacterial meningitis, more commonly observed in individuals with compromised immune systems. Furthermore, when caused by a gram-negative organism, these cases have largely been reported as ventricular catheter-related. This case report presents a unique instance of pyogenic ventriculitis caused by *Escherichia coli* (*E. coli*) in a 48-year-old female, wherein a sigmoid colon fistula led to vertebral osteomyelitis and subsequent inoculation of the cerebrospinal fluid. The patient presented with chronic back pain and a one-week history of fever, nausea, vomiting, and generalized weakness. In addition to these symptoms, the patient also had a significant medical and surgical history, with cervical cancer and pelvic radiation exposure being two notable features. Although antibiotic therapy was provided due to *E. coli* bacteremia, a decision to pursue supportive care was made given the presence of extensive comorbidities. Therefore, the purpose of this case report is not only to discuss an atypical case of meningitis/ventriculitis, but also to emphasize diagnostic findings and treatment options that could have been used for our patient if circumstances permitted.

## Introduction

The central nervous system (CNS) functions as the master regulator of vital bodily processes. Within the central nervous system (CNS), cerebrospinal fluid (CSF) circulates, providing essential nutrients to neurons and glial cells, thereby supporting their proper function. CSF is primarily produced by the choroid plexus located in the lateral ventricles [1]. From there, CSF flows through the interventricular foramina into the third ventricle, and then continues through the cerebral aqueduct to reach the fourth ventricle. At this point, some of the fluid exits the ventricular system via the median foramen of Magendie and the paired lateral foramina of Luschka, entering the subarachnoid space. A portion also flows into the central canal of the spinal cord [1]. This continuous flow highlights the close interconnection between the brain’s ventricular system and the spinal canal.

Ventriculitis often presents with symptoms resembling meningitis, including headache, nausea, and neurological disturbances such as nuchal rigidity, seizures, and altered mental status [2]. This condition typically arises as a complication of various etiologies like meningitis, brain abscesses, traumatic brain injury, CSF leaks, intrathecal chemotherapy, or neurosurgical procedures [3]. The presence of suppurative fluid within the ventricles, occurring as a complication of bacterial meningitis, is termed 'pyogenic ventriculitis' [4]. This condition is linked to a high mortality rate, approximately 30%, and has a 60% incidence of neurological sequelae [3]. Thus, early recognition of signs and symptoms is vital for effective treatment.

In this report, we will present a rare case of pyogenic ventriculitis originating from a sigmoidal fistula attached to the L5-S1 vertebrae. Subsequent vertebral osteomyelitis led to meningitis, which was further complicated by ventriculitis. In this report, we aim to discuss potential treatment options and key imaging findings to facilitate earlier recognition of this atypical infection.

## Case presentation

This case involves a 48-year-old female with a complex medical history, including stage IIIC squamous cell carcinoma of the cervix (cT2bN1), initially treated with chemoradiation therapy in 2020. Following recurrence, she underwent treatment with cisplatin and radiation in 2022. The patient also has a documented history of an incarcerated hernia and suspected bowel perforation, which necessitated an exploratory laparotomy, extensive enterolysis, two small bowel resections with reanastomosis, hernia repair, anterior exenteration accompanied by a vertical rectus abdominis myocutaneous (VRAM) flap, ileal conduit formation, and an omental J flap.

**Figure 1:**
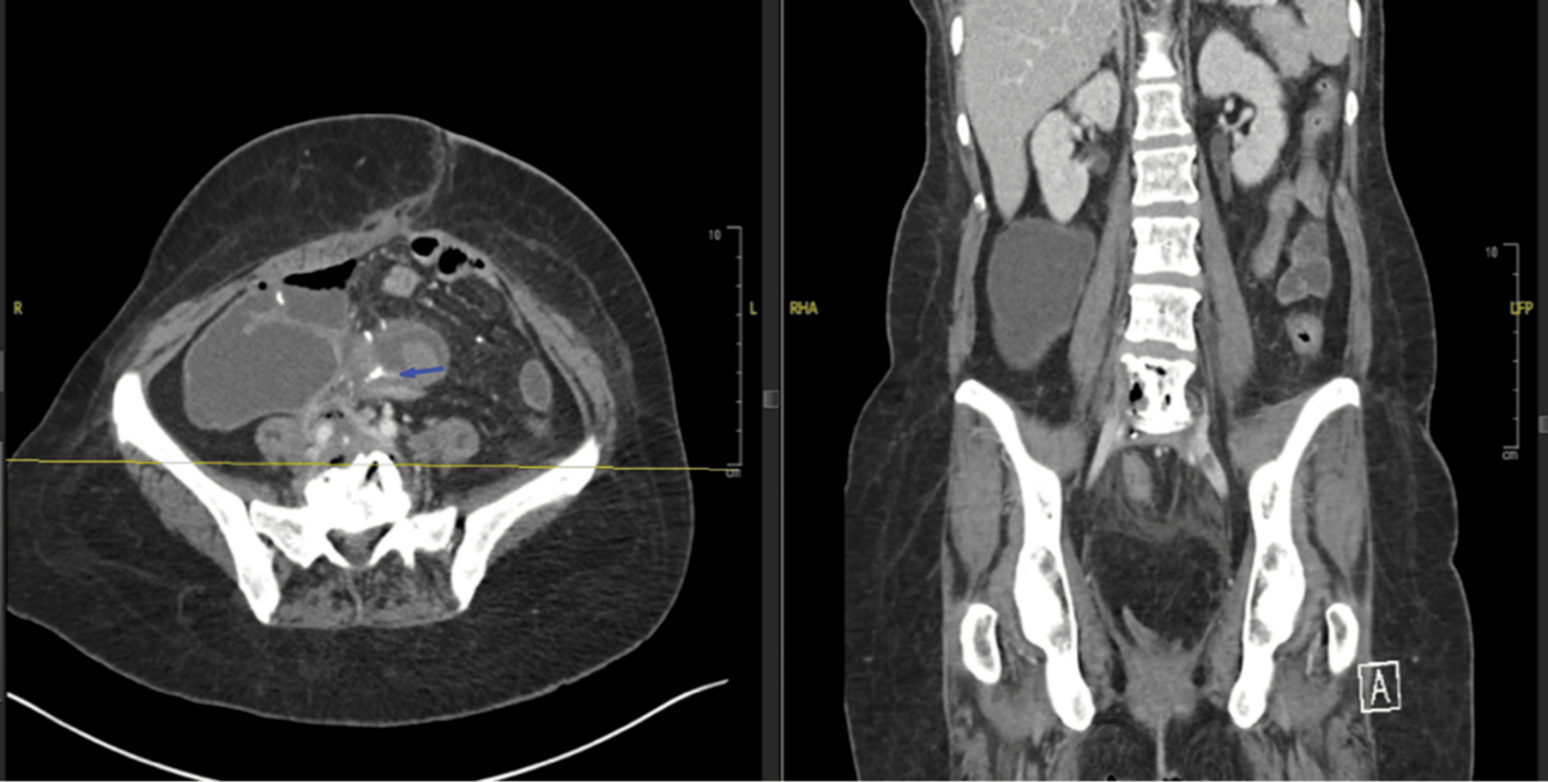
CT hyperdense signal pointed by the blue arrow indicated the sigmoidal fistula (Left). Cortical erosion was along the anterior surface of the L5 vertebral body (Right).

The patient presented to the Moffitt Cancer Center with complaints of exacerbated chronic back pain, in addition to a one-week history of subjective fever, nausea, vomiting, and generalized weakness. Upon admission, the patient was afebrile, with vital signs recorded as follows: blood pressure 124/80 mm Hg, pulse 79 beats per minute, respiratory rate 13 breaths per minute, and oxygen saturation of 96% on room air. Blood and urine cultures were subsequently obtained, indicating the presence of *E. coli* in both samples, prompting the initiation of intravenous ceftriaxone, 2 grams daily, due to *E. coli* bacteremia. A computerized tomography (CT) scan showed inflammation of the sigmoid colon, particularly in proximity to the L5-S1 region, as well as cortical erosion along the anterior surface of the L5 vertebral body, suggesting possible fluid extension along the anterior surface of L5-S1, with an epidural abscess not being excluded (Figure 1). Notably, air was noted with imaging, which was concerning for infection involving a gas-producing organism.

**Figure 2:**
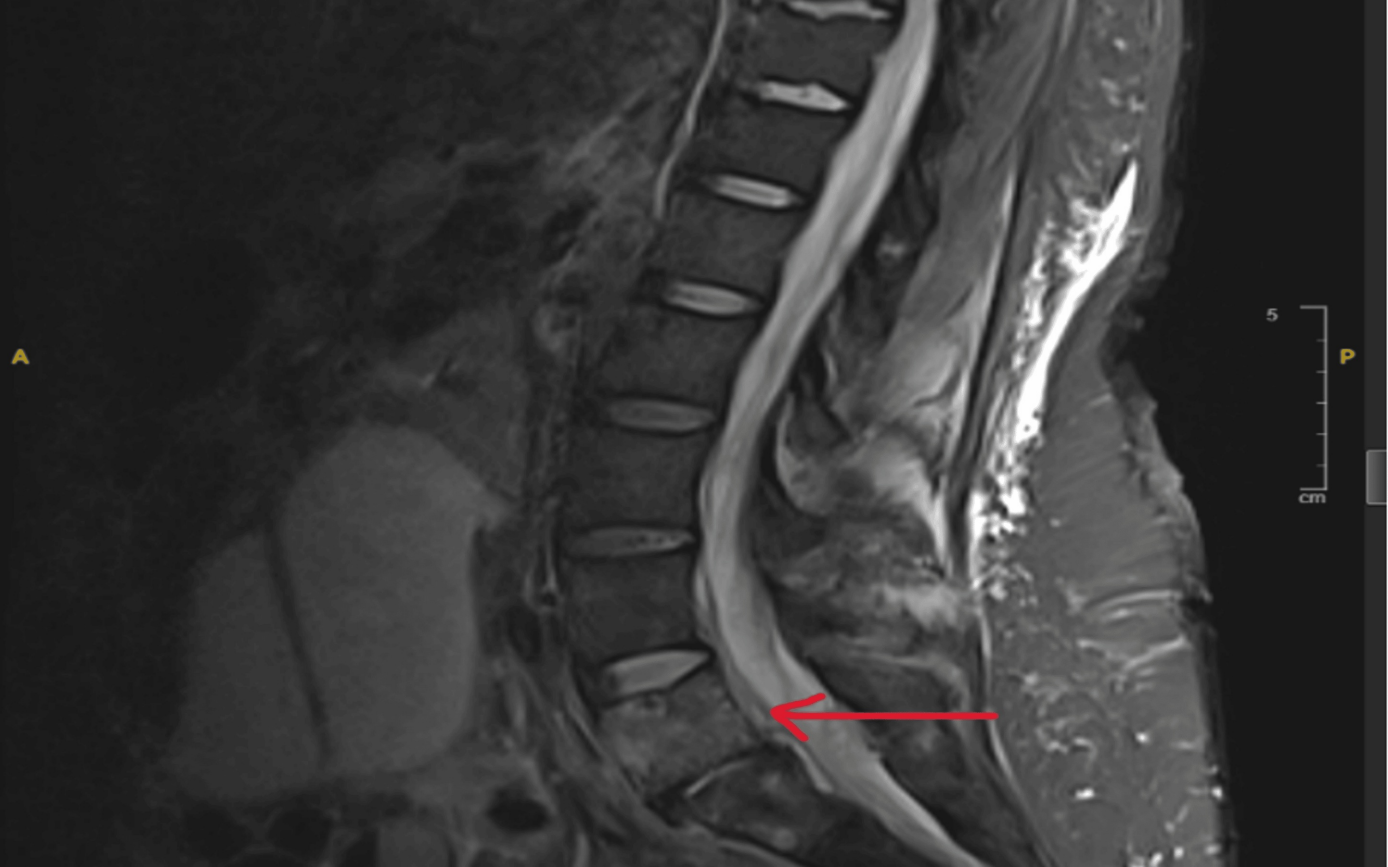
MRI of the spine showing L5-S1 discitis concerning for potential osteomyelitis.

**Figure 3:**
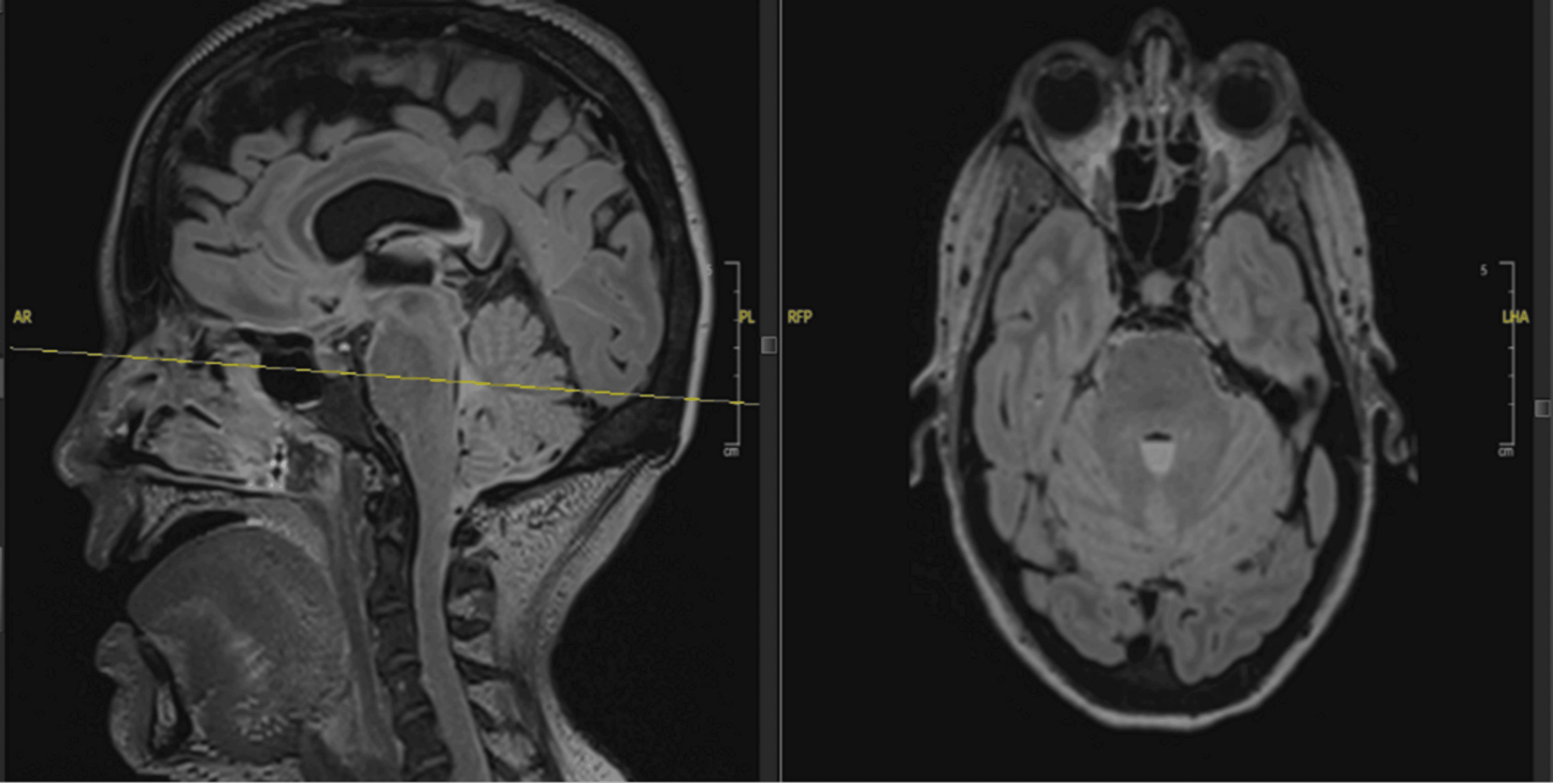
The sagittal plane (left) and transverse plane (right) of the MRI revealed ventriculitis with fluid level in the fourth ventricle.

**Figure 4:**
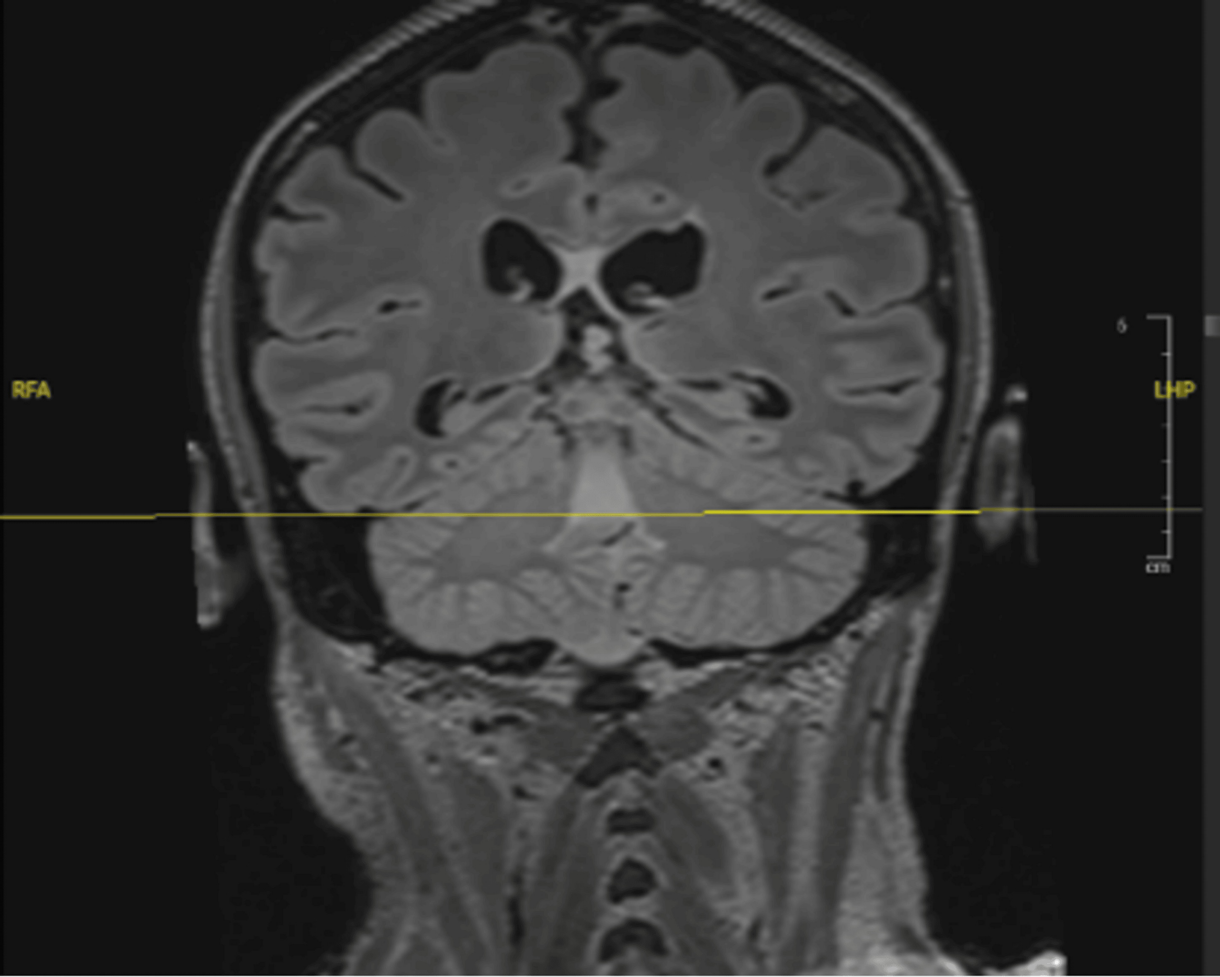
The coronal plane of the MRI revealed ventriculitis with fluid level in the fourth ventricle.

Following these findings on CT imaging, an MRI of the brain and lumbar spine was also conducted with and without contrast. MRI of the spine revealed an intra-abdominal abscess, sigmoid bowel fistulization to the L5-S1 vertebrae, and inflammatory changes suggestive of L5-S1 acute on chronic osteomyelitis and discitis (Figure 2). The MRI of the brain revealed a fluid level within the fourth ventricle, indicating possible blood or pus formation (Figures 3, 4). With CSF inoculation as a concern, a lumbar puncture was attempted; however, with an opening pressure of less than 9 cm H_2_0, no fluid could be obtained. Given the presence of a colonic fistula along with *E. coli* bacteremia, this organism became the focus of treatment. In addition to ceftriaxone, given findings indicative of extensive infection, intravenous metronidazole (500mg, three times daily) was added to the antibiotic regimen. However, considering the patient’s extensive medical and surgical history, the medical team engaged in a comprehensive discussion regarding treatment options, carefully weighing the associated risks and benefits. Ultimately, a consensus was reached with the patient’s family to transition to supportive care.

## Discussion

CNS infections caused by Gram-negative bacilli are rare, accounting for 8.7% of all meningitis cases. Among these, *E. coli* is seen in 42% of infections [5]. *E. coli* is a well-known pathogen for meningitis in neonates; however, when this pathogen causes meningitis in the adult population, it is typically associated with recent neurosurgical procedures, head trauma, or infections disseminated from the urinary or gastrointestinal systems [6]. In terms of severity, Gram-negative bacterial meningitis was reported to have a 53% mortality rate in the adult population, with *E. coli *accounting for 38% of these deaths [7]. Another study reported a 36% mortality rate among patients with *E. coli *meningitis in patients over the age of 16. This rate was significantly higher than the 20% mortality rate associated with pneumococcal meningitis and the 7% for meningococcal meningitis [8]. These statistics highlight the severity of *E. coli* meningitis, underscoring the importance of rapid identification and treatment for optimal patient outcomes.

In addition to meningitis caused by an atypical bacterial pathogen, this case also features the development of pyogenic ventriculitis (PV) as a rare complication of bacterial meningitis. A major risk factor to consider when encountering this complication in the adult population is compromised immunity observed in conditions such as cancer, HIV, diabetes, and alcohol use disorder. Common pathogens to consider in cases of pyogenic ventriculitis include *Streptococcus* species,* Staphylococcus *species, and Gram-negative bacilli [2]. However, it is important to note that literature on cases caused by *E. coli* remains extremely limited, thereby highlighting the significance of this case.

Another distinct feature of this case involves the unconventional route of the meningeal infection. Having been exposed to multiple rounds of pelvic radiation, first in 2020 and again in 2022, it is reasonable to attribute the development of a fistula as the source of infection, connecting the sigmoid colon to the L5-S1 vertebrae. Furthermore, vertebral osteomyelitis is a predisposing factor in only 0.5% of meningitis cases [9]. To the best of our knowledge, this is the first reported case of extensive spread from a colonic fistula to the CSF via vertebral osteomyelitis. When meningitis and vertebral osteomyelitis present concurrently, it raises the classic clinical dilemma of determining which pathology preceded the other. For this case report, we have a clear answer.

In terms of making a diagnosis, CSF analysis is crucial for identifying the pathogenic cause of infection. Unfortunately, we could not obtain from our patient. However, with the use of radiological imaging, complications, such as ventriculitis, can be appreciated and are suggestive of infection. When it comes to identifying ventriculitis, the literature is limited. Magnetic resonance imaging (MRI) typically shows debris layering in the ventricles, which appears hyperintense on T1-weighted imaging and hypointense on T2-weighted imaging in up to 94% of ventriculitis cases [2,10]. Other findings include periventricular hyperintensity (seen in 78% of cases on fluid-attenuated inversion recovery [FLAIR] sequences) and ependymal enhancement (seen in 64% of cases) [2,11,12]. Diffusion-weighted imaging (DWI) can also be useful in detecting pus in the ventricular system [12]. Notably, our patient’s MRI did not reveal these findings. Instead, a "fluid-fluid level" in the fourth ventricle was seen, indicating pus or blood within the ventricles. Additionally, there was a lack of CSF signal suppression on FLAIR in the posterior fossa, likely due to the increased protein content associated with bacterial infection. These findings provide potential alternative clues that can be used to help identify pyogenic ventriculitis more rapidly if other well-documented presentations cannot be observed.

Aside from surgical correction of the sigmoidal fistula, a treatment timeline with antibiotic therapy for each presenting complication is also necessary. The recommended duration of treatment for osteomyelitis in adults is four to six weeks of antibiotic therapy with a combination of vancomycin plus a third-generation cephalosporin or a beta-lactam/beta-lactamase inhibitor [13]. This brings up an important consideration: for isolated meningitis, a minimum of a 14-day course of antibiotics is recommended for the most common causative pathogens (Group B *Streptococcus* and *Streptococcus pneumoniae*). This recommendation is extended to a 21-day course for gram-negative enteric bacteria, such as *E. coli *[14]. However, even with this extension, this does not provide an adequate duration of antibiotic therapy for osteomyelitis (4-6 weeks) or pyogenic ventriculitis, which literature has demonstrated requires 6 to 12 weeks of antibiotic therapy [4]. This underscores the importance of accurately identifying the underlying cause of CNS infection and utilizing laboratory findings and imaging studies effectively for timely diagnosis.

In terms of treatment recommendations for pyogenic ventriculitis, particularly in cases caused by Gram-negative organisms such as *E. coli*, the literature is limited, as most research focuses on precise guidelines for the management of ventricular-catheter-related and healthcare-associated ventriculitis. Nonetheless, current guidelines recommend empiric therapy that provides coverage for both gram positive and gram negative microbes. Specifically, vancomycin combined with an antipseudomonal beta-lactam (e.g., cefepime, ceftazidime, or meropenem) is recommended for the treatment of healthcare-associated ventriculitis [12]. Once the causative organism is identified and susceptibility is determined, treatment should be adjusted with the use of antibiotic therapy, which should demonstrate adequate CNS penetration. If systemic therapy alone is insufficient, intraventricular antibiotic administration is recommended. Commonly used antibiotics include aminoglycosides, colistin, daptomycin, tigecycline, and vancomycin [2]. However, research has begun to evaluate the role of lavage and drainage along with the addition of antibiotics for treating ventriculitis. Traditionally, infected CSF is drained via external ventricular drainage (EVD) or a lumbar drain. However, these methods often show reduced effectiveness in patients with severe ventriculitis, especially when factors such as pus/flakes exist within the CSF from active infection. In such cases, lavage may offer a potential benefit by removing this ventricular empyema, preventing adhesion and interference with the delivery of antibiotics to their intended site [15].

A study comparing lavage with conventional drainage (via an external ventricular drain) found that the lavage group had a significantly lower mortality rate (25% vs. 52.9%) and spent fewer days in the hospital [16]. Another study reported a 100% cure rate with lavage, as none of the patients had positive CSF cultures after the procedure. In addition to this cure rate, they found that treatment with lavage after 7-10 days of standard intravenous (IV) and intrathecal (IT) antibiotics was ideal for patient care [15]. While this study had a small sample size and calls for further research with larger groups, these promising results support the idea that lavage can be an effective approach for the rapid and successful treatment of ventriculitis.

After adequate treatment, another barrier to complete therapy is patient loss due to the necessity for long-term follow-up, as the risk for recurrent ventriculitis and hydrocephalus remains elevated after completion of antibiotic therapy. Research has demonstrated that the ventricles and choroid plexus can serve as reservoirs for infection, even when the lumbar puncture yields sterile cultures [2]. Thus, to achieve comprehensive care, the treatment of pyogenic ventriculitis truly requires extensive monitoring even after prolonged antibiotic therapy.

## Conclusions

This case report aims to achieve two objectives: first, to highlight an atypical pathway resulting in pyogenic ventriculitis caused by an uncharacteristic pathogen, and second, to draw attention to potential diagnostic features and treatment options that may improve patient outcomes. This case illustrates the severity and uniqueness of* E. coli *meningitis complicated by ventriculitis, as well as the necessity for achieving a rapid diagnosis and aggressive treatment with antibiotics in addition to invasive surgical interventions. In similar cases, prompt identification of the causative organism is essential to guide appropriate management and improve outcomes. Standard practice involves a lumbar puncture; however, when unobtainable, as in our case, one could resort to imaging for diagnostic clues. Once ventriculitis is identified, empiric treatment providing coverage for both Gram-positive and Gram-negative organisms is essential. Subsequently, based on clinical improvement, one should consider intraventricular antibiotics in conjunction with lavage and drainage. Given our patient's extensive medical and surgical history, the healthcare team determined that attempting to address the primary source of the CNS infection posed more risk than benefit. As a result, the decision was made to forgo further intervention in favor of supportive care.
